# Experience of infertility-related stigma in Africa: a systematic review and mixed methods meta-synthesis

**DOI:** 10.1093/inthealth/ihaf060

**Published:** 2025-05-27

**Authors:** Emmanuel Ekpor, Samuel Sanaa Brobbey, Cynthia Yaba Kumah, Samuel Akyirem

**Affiliations:** School of Nursing and Midwifery, University of Ghana, Accra, Ghana; School of Psychology, Deakin University, Geelong, Victoria, Australia; African Centre of Excellence for Public Health and Toxicological Research, University of Port Harcourt, Port Harcourt, Nigeria; Department of Midwifery, Kwame Nkrumah University of Science and Technology, Kumasi, Ghana; Yale School of Nursing, Yale University, New Haven, CT, USA

**Keywords:** Africa, infertility, stigma, systematic review

## Abstract

Infertility is a significant reproductive health issue with high prevalence rates in Africa, where it is often stigmatized. This systematic review characterizes the experience of infertility stigma in Africa. A systematic search of relevant studies was conducted across PubMed, MEDLINE, CINAHL, PsycINFO, Global Health, Scopus and Web of Science, covering publications from their inception to March 2025. The search incorporated subject headings and keywords related to ‘infertility’ and ‘stigma’ in combination with terms specifying all African countries. A mixed methods approach was employed for data analysis, using the convergent thematic QUAL synthesis method. A total of 1987 records were identified, with 48 studies ultimately meeting the inclusion criteria. The analysis revealed four overarching themes in the experience of infertility stigma: mechanisms of stigma—factors that drive or perpetuate stigma surrounding infertility; stigma marking—the social markers and identifiers that distinguish individuals as stigmatized; manifestations of stigma—the varied forms through which stigma is expressed; and consequences—the psychological, behavioural, relational and health-related repercussions for individuals experiencing infertility stigma. Infertility stigma was widespread, with a prevalence as high as 64%. However, no interventions were developed and implemented to mitigate this issue. Infertility stigma in Africa is deeply embedded within cultural norms and social structures, significantly impacting the lives of those affected. This review emphasizes the critical need for culturally tailored interventions to alleviate stigma and enhance access to reproductive health services.

## Introduction

Infertility is a pressing reproductive health issue, affecting about 17.5% of individuals over their reproductive lifespan.^1^ Clinically it is defined as the inability to conceive after at least 12 months of regular, unprotected sexual intercourse.^2^ However, infertility extends far beyond its medical dimensions; it is intricately woven into the cultural and social fabric of societies, influencing norms surrounding reproduction and parenthood.^3^ In Africa, where infertility prevalence is estimated at 49%,^4^ the condition carries profound social consequences, often leading to stigma and discrimination.

In many African societies, parenthood is central to personal identity and social belonging, with motherhood regarded as a marker of success in marriage.^5^^,^^6^ Infertility disrupts these cultural expectations and women, in particular, bear the brunt of stigma. They may be blamed for their condition, labelled as incomplete and subjected to ridicule or abandonment.^6^^,^^7^ Marital instability is common, as infertile women often face rejection or polygamous arrangements initiated by their partners in pursuit of biological children.^8^^,^^9^ Men, though less frequently stigmatized, may experience silent suffering due to societal expectations of masculinity and virility.^10^^,^^11^ Religious ideologies further exacerbate infertility stigma. In some communities, infertility is viewed as a curse or spiritual failing, leading to marginalization. Women unable to conceive may be accused of bringing misfortune to their families, intensifying psychological distress.^10^^,^^12^ Research highlights the emotional toll, with Ochieng^13^ describing infertility as ‘something so serious that one would try so hard to overcome’ and Silva^14^ portraying it as ‘a sad, regretful handicap’ for men and ‘a tragedy that [a woman] cannot escape biologically nor socially’. These narratives illustrate how infertility is perceived not just as a medical condition but as a profound social crisis.

Infertility stigma is indeed a significant burden in Africa and is estimated to affect about 64% of individuals.^15^ Several studies have explored various dimensions of this issue, including its manifestations, drivers and consequences for those affected. However, existing evidence is fragmented, lacking a consolidated framework to examine the diverse and complex ways infertility stigma is experienced across the continent. This systematic review seeks to address this gap by synthesizing empirical findings from studies on infertility stigma in Africa. By characterizing these experiences, this review aimed to contribute to the development of a framework that can guide future research, inform policy formulation and shape interventions aimed at alleviating the negative impact of infertility stigma on individuals and communities.

## Methods

### Design and search strategy

A systematic review was conducted following the Preferred Reporting Items for Systematic Reviews and Meta-Analyses (PRISMA) guideline^16^ and was prospectively registered on PROSPERO (CRD42024579404). Our approach integrated both quantitative and qualitative evidence to gain a holistic understanding of infertility-related stigma in Africa. Searches for relevant studies were executed on PubMed, MEDLINE, CINAHL, PsycINFO, Global Health, Scopus and Web of Science. Our search was initially conducted in June 2024 and was updated in March 2025. The search strategy was built on three concepts—‘infertility’, ‘stigma’, and ‘Africa’—and did not apply any limits or filters. Database-specific combinations of the indexed terms and keywords of these concepts were used to form the search string. The Boolean operators ‘OR’ and ‘AND’ were applied appropriately. To ensure exhaustiveness of the search, forward and backward citation tracking of studies was done. This included reviewing the reference lists of all included studies as well as employing Google Scholar's citation search tool to identify studies that cited key articles. The full details of the search strategy are provided in the supplementary file.

### Study selection

To be included in this review, we considered primary qualitative, quantitative or mixed methods research providing empirical data on infertility stigma in Africa. Although we prioritized lived experience of stigma in people with infertility, studies that explored stigma encounters from the perspectives of key informants, including partners, healthcare providers, family members and communities, were also included in this review. This multifaceted approach enabled a more comprehensive understanding of how infertility stigma is experienced and perpetuated within different contexts in Africa.

We excluded studies that were review articles, from grey literature sources (e.g. abstracts, conference proceedings) or were published in a non-English language. Grey literature was excluded due to the potential lack of peer review and methodological rigor, which could affect the reliability and validity of the findings. Non-English-language studies were excluded due to translation barriers, which could lead to incomplete or inaccurate data extraction and synthesis.

The selection of eligible studies was performed using Rayyan (Rayyan, Cambridge, MA, USA). This was done in two stages: first, by reviewing the titles and abstracts of the retrieved articles, and then by examining their full text. Two independent reviewers conducted the study selection. Discrepancies were resolved through discussion and a third reviewer was consulted if consensus could not be reached.

### Quality assessment

The Mixed Method Appraisal Tool (MMAT) version 2018 was used in assessing the quality of the included studies.^17^ The MMAT was used because it is the recommended assessment tool for mixed methods reviews.^18^ This tool includes seven questions (two general screening and five study design–specific questions), each of which is rated on a 3-point scale: yes, no or can't tell. to calculate a quality score, responses of ‘yes’ are assigned a value of 1, while both ‘no’ and ‘can't tell’ receive a score of 0.

### Data extraction and synthesis

A data extraction matrix, previously used in diabetes stigma research in Africa, was adapted for this study.^19^ The information gathered included authors, year of publication, country, study design, sample characteristics and findings related to infertility stigma. Convergent thematic QUAL synthesis was used to transform both quantitative and qualitative data into themes, enabling the integration of stigma-related findings from the included studies.^18^ Themes were developed using the inductive (theory-building) approach. Each study was thoroughly read and reviewed independently by at least two reviewers. Subsequently, data were coded and key themes related to infertility stigma were identified through NVivo 14 (Lumivero, Denver, CO, USA). Reviewers engaged in discussions to compare the emergent themes across studies. Discrepancies were resolved through consensus.

## Results

**Figure 1.**
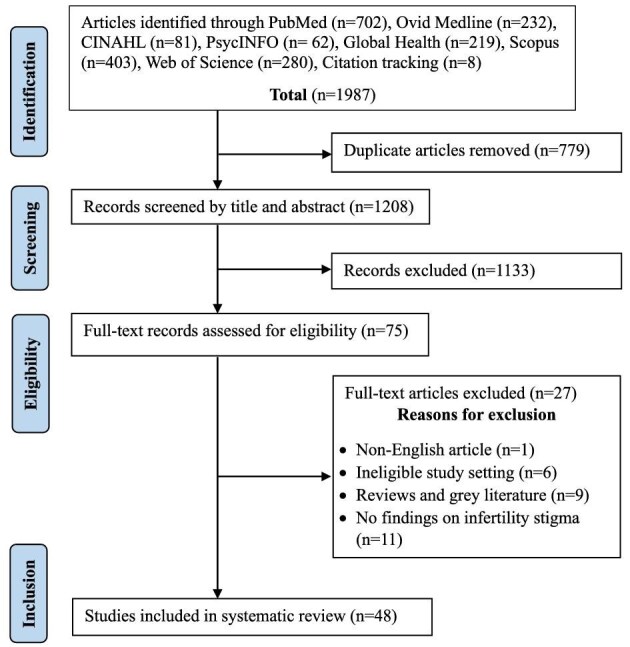
PRISMA diagram of the screening process.

A total of 1987 records were retrieved, including 1807 from our initial search. Duplicates of 779 were identified and removed. The remaining articles were screened based on their titles and abstracts, leading to the exclusion of 1133 studies. Subsequently, 75 full-text articles were thoroughly assessed and 48 (including 2 articles from the updated search) were deemed eligible for inclusion. Figure 1 provides a detailed overview of the study selection process, including the reasons for the exclusion of articles following full-text screening.

### Characteristics of included studies

The reviewed studies were published between 2002 and 2025, with the majority (n=33) published in the last decade. Participants primarily comprised individuals with infertility, although some studies included key informants, such as healthcare providers, community and religious leaders and traditional healers. The duration of infertility among participants ranged from 1 to 27 years. Research was conducted in 15 countries, with Ghana contributing the most studies (n=18),^5^^,^^6^^,^^10^^,^^12^^,^^15^^,^^20–32^ followed by Nigeria (n=6),^8^^,^^33–37^ Ethiopia (n=3),^7^^,^^38^^,^^39^ South Africa (n=3),^40–42^ Zimbabwe (n=3),^11^^,^^43^^,^^44^ Cameroon (n=2),^9^^,^^45^ Gambia (n=2),^46^^,^^47^ Malawi (n=2),^48^^,^^49^ Kenya (n=2),^13^^,^^50^ Uganda^51^^,^^52^ and one study each from Mali,^53^ Morocco,^54^ Rwanda,^55^ Senegal^56^ and Tanzania.^57^ Most studies employed qualitative designs, while 8 used quantitative (n=5) or mixed methods (n=3) approaches. All the quantitative studies were cross-sectional, and only one explicitly used a scale designed for infertility stigma, while others adapted scales developed to measure stigma in relation to other conditions (Table 1).

**Table 1.** Characteristics of included studies.

Author (year)	Country	Study type	Data collection/instrument	Population of interest	Years infertile	Quality score
Kyei et al. (2024)^21^	Ghana	Quantitative	Infertility Stigma Scale (ISS) questionnaire	Women with infertility (n=302)	NR	5
Naab et al. (2013)^24^	Ghana	Quantitative	5-item Stigma Scale for Receiving Psychological Help	Women (married and unmarried) with infertility (n=203)	NR	4
Donkor and Sandall (2007)^15^	Ghana	Quantitative	3-item stigma scale developed originally for persons with stroke	Women (n=615) with infertility	NR	5
Van Rooij et al. (2021)^27^	Ghana	Quantitative	Donkor and Sandal (2007) 3-item stigma scale developed originally for persons with stroke	Men (n=11) and women (n=38) with infertility	Mean 7.61	4
Anokye et al. (2017)^32^	Ghana	Quantitative	Self-developed questionnaire	Couples with infertility (n=100)	NR	4
Kyei et al. (2021)^20^	Ghana	Qualitative	Interviews	Men (n=6) and women (n=12) with infertility	2–27	7
Tabong and Adongo (2013)^22^	Ghana	Qualitative	In-depth interviews, focus group discussions, and key informant interviews	Childless couples (n=15), couples with children (=45), and key informant (n=8)	NR	7
Tabong and Adongo (2013)^23^	Ghana	Qualitative	In-depth interviews, focus group discussions and key informant interviews	Childless couples (n=15), couples with children (n=45) and key informant (n=8)	NR	7
Naab and Kwashie (2018)^25^	Ghana	Qualitative	Semi-structured interview	Married men with infertility (n=12)	NR	7
Naab et al. (2019)^8^	Nigeria	Qualitative	Semi-structured interview	Married women with infertility (n=12)	NR	7
Donkor et al. (2017)^26^	Ghana	Qualitative	In-depth interviews	Women with infertility (n=14)	2–13	7
Kuug et al. (2023)^10^	Ghana	Qualitative	Semi-structured interview	Male (n= 8) and female (n=7) partners with infertility	5–26	7
Azize Diallo et al. (2024)^12^	Ghana	Qualitative	In-depth interviews	Couples with infertility (n=10)	3–9	7
Fledderjohann (2012)^5^	Ghana	Qualitative	Semi-structured interview	Women (married and unmarried) with and without infertility (n=107)	NR	7
Annan-Frey et al. (2023)^28^	Ghana	Qualitative	Semi-structured interview	Women with infertility (n=19)	1.5–15	7
Ofosu-Budu and Hanninen (2020)^6^	Ghana	Qualitative	In-depth interviews	Married women with infertility (n=30)	NR	7
Adane et al. (2024)^7^	Ethiopia	Qualitative	In-depth interviews, focus group discussions and key informant interviews	Women with infertility (n=15) and key informants (n=15)	NR	7
Adane et al. (2024)^38^	Ethiopia	Qualitative	In-depth interviews	Women with infertility (n=15)	NR	7
Dierickx et al. (2018)^46^	Gambia	Qualitative	Interviews, group discussions, participant observation and informal conversations	Women with infertility (n=33)	NR	7
Dierickx et al. (2021)^47^	Gambia	Qualitative	In-depth interviews	Men with infertility (n=13)	NR	7
Dierickx (2022)^56^	Senegal	Qualitative	Interviews, participant observation and informal conversations	Women with infertility (n=11)	NR	7
Mussie (2017)^39^	Ethiopia	Qualitative	In-depth interviews, key informant interviews and case histories	Men (n=11) and women (n=15) with infertility, and key informant (n=4)	NR	6
Moyo (2013)^11^	Zimbabwe	Qualitative	In-depth interviews, key informant interviews and focus group discussions	Men (n= unspecified)	NR	6

**Table 1.** Continued

Author (year)	Country	Study type	Data collection/instrument	Population of interest	Years infertile	Quality score
Bornstein et al. (2020)^48^	Malawi	Qualitative	Focus group discussions	Men (n=51) and women (n=53)	NR	7
Weinger (2009)^45^	Cameroon	Qualitative	Interviews	Women with infertility (n=5)	NR	4
Esan et al. (2022)^33^	Nigeria	Qualitative	Semi-structured interviews	Men (n=2) and women (n=13) with infertility	NR	7
Hess et al. (2018)^53^	Mali	Mixed	Semi-structured interviews (for qualitative data)	Women with infertility (n=58)	NR	5
Hiadzi (2022)^29^	Ghana	Qualitative	In-depth interviews	Women with infertility (n=35)	NR	7
Dyer et al. (2002)^40^	South Africa	Qualitative	In-depth interviews	Women with infertility (n=30)	1–15	7
Dyer (2004)^41^	South Africa	Qualitative	In-depth interviews	Men suffering from couple infertility (n= 27)	NR	7
Hollos and Whitehouse (2014)^34^	Nigeria	Qualitative	In-depth interviews	Women with and without infertility (n=50)	NR	3
Hollos and Larsen (2008)^57^	Tanzania	Qualitative	In-depth interviews	Women with and without infertility (n=50)	NR	3
Arhin et al. (2022)^30^	Ghana	Qualitative	Telephone interviews and key informant interviews	Men (n=6) and women (n=14) with infertility and healthcare personnel (n=8)	NR	7
Elwell (2022)^49^	Malawi	Qualitative	Semi-structured interview and focus group discussions	Women (n=78), healthcare personnel (n=12) and community leaders (n=32)	NR	6
Mabasa (2002)^42^	South Africa	Qualitative	Semi-structured interviews	Men (n=30) and women (n=46)	NR	7
Okantey et al. (2021)^31^	Ghana	Qualitative	Interviews	Clients who have accessed assisted reproductive technology (n=16) and healthcare personnel (n=4)	NR	7
Olowokere et al. (2022)^35^	Nigeria	Mixed	In-depth interviews (for qualitative data)	Women with infertility (n=152)	1–20 (mean 4.1)	5
Asiimwe et al. (2022)^51^	Uganda	Qualitative	In-depth interviews	Women with infertility (n=15)	2–≥16	7
Dimka and Dein (2013)^36^	Nigeria	Qualitative	Semi-structured interviews and focus group discussions	Men (n=6) and women (n=8) with and without infertility and key informants (n=14)	NR	7
Nieuwenhuis et al. (2009)^37^	Nigeria	Qualitative	In-depth interviews and focus group discussions	Men (n=7) and women (n=8) with infertility, community members (n=42), healthcare personnel and traditional healers (n=13)	NR	7
Dhont et al. (2011)^55^	Rwanda	Mixed	Focus group discussions (for qualitative data)	Couples with infertility (n=312) and those fertile (n=312)	1–≥8	3
Nguimfack et al. (2016)^9^	Cameroon	Qualitative	Semi-structured interview	A premenopausal woman with infertility	NR	7
Njogu et al. (2022)^50^	Kenya	Qualitative	Semi-structured interview	Women with infertility (n=33)	2–14	7
Mashaah et al. (2024)^43^	Zimbabwe	Qualitative	Interviews	Women with infertility (n=5)	NR	7
Ingwani et al. (2021)^44^	Zimbabwe	Qualitative	Interviews	Men (n=5) and women (n=5)	NR	6
Ochieng’ (2020)^13^	Kenya	Qualitative	In-depth interviews and focus group discussions	Catholic childless couples and key informants (priests, catechists and Christian community leaders)	NR	6
Pratt et al. (2025)^52^	Uganda	Qualitative	In-depth interviews	Men (n=30) with comorbid HIV and infertility and female partners (n=10)	NR	7
Benbella et al. (2025)^54^	Morrocco	Qualitative	In-depth interviews	Couples with infertility (n=28), healthcare personnel (n=6) and policy stakeholders (n=5)	NR	7

### Quality of included studies

The quality assessment scores for the included studies ranged from 4 to 5 for quantitative studies, 3 to 7 for qualitative studies and 3 to 5 for mixed methods studies (Table 1). As detailed in the supplementary file, the quantitative studies exhibited methodological weaknesses, including non-random participant selection, lack of clarity regarding the representativeness of the target population and inadequate reporting on non-response bias. For the qualitative studies, key limitations involved the insufficient detail provided on data analysis processes, which hindered the ability to ascertain whether the findings were sufficiently grounded in the data. In the mixed methods studies, there were deficiencies in adequately addressing the discrepancies and inconsistencies between the quantitative and qualitative results. Additionally, the individual qualitative and quantitative components did not fully adhere to established methodological standards.

### Findings from meta-synthesis

**Figure 2.**
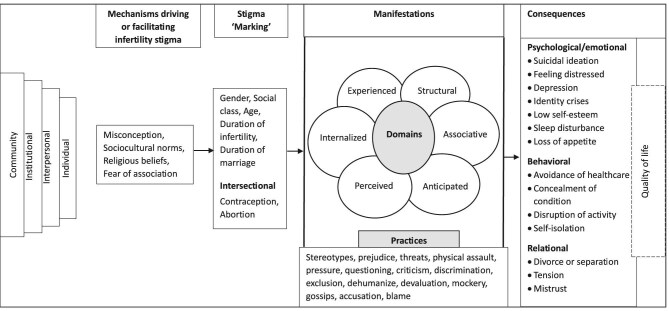
A framework for infertility stigma in Africa.

Four major themes emerged from our thematic analysis: mechanisms driving or facilitating infertility stigma, stigma marking, manifestations and consequences. These themes were incorporated into a framework that illustrates and characterizes the experience of infertility stigma in Africa (Figure 2).

### Mechanisms driving or facilitating infertility stigma

The stigma surrounding infertility in Africa is deeply embedded within a nexus of entrenched sociocultural narratives, religious ideologies and institutionalized beliefs. Misconceptions about the aetiology of infertility were pervasive, with many communities framing it through moralistic and supernatural lenses. Infertility was frequently attributed to actions deemed socially or morally unacceptable, such as prior abortions^9^^,^^20^^,^^31^^,^^35^^,^^36^^,^^40^ or the use of contraceptives.^6–8^^,^^23^^,^^46^ Beyond these, it was often construed as divine punishment,^9^^,^^10^^,^^31^^,^^44^^,^^48^ the result of witchcraft^10^^,^^22^^,^^36^^,^^43^^,^^44^^,^^47^^,^^51^ or other metaphysical phenomena.^5^^,^^43^ These socially constructed explanations obscured biomedical understandings of infertility, situating affected individuals as morally culpable or inherently flawed. For instance, a participant in one study revealed, ‘everyone says I should suffer the consequences of my mistakes [alleged abortions]’,^9^ reflecting the widespread belief that infertility is self-inflicted.

Moreover, procreation in many African societies was not only seen as a biological process, but as a sociocultural obligation that upheld family lineage, inheritance rights and social standing. Individuals unable to conceive faced severe social scrutiny and were often labelled as ‘failures’.^37^^,^^40^^,^^49^ In some communities, childbearing is tied to identity, with the inability to have a child undermining one's status. This sentiment was reflected in settings where those who could not bear children were seen as failing to fulfil the criteria for ‘mature’, ‘full’, ‘complete’, or ‘real’ manhood or womanhood,^7^^,^^10^^,^^25^^,^^39^^,^^47^ and to an extent, some were ‘not considered to be a person’.^41^ Various practices embedded in the cultural fabric of certain African societies reinforce the experience of stigma. In South Africa, for instance, brides are required to wear a ‘doek’ (scarf) until they have children,^40^ creating easily identifiable markers of their fertility status and thus increasing their exposure to stigma. In Ghana, even after death, cultural rituals perpetuate stigma: men who die without fathering children are dressed in women's clothing, reinforcing the notion that masculinity and worth are inextricably linked to fatherhood.^10^ Fear also plays a significant role in perpetuating infertility stigma. Infertility is often perceived as a curse, leading to social distancing as people fear being associated with the ‘cursed’ status of childlessness.^10^

### Stigma marking

The concept of ‘stigma marking’, wherein infertility stigma is applied to individuals based on distinguishing traits or characteristics, is evident across various African contexts. Gender, particularly being a woman, often serves as a primary marker for infertility stigma. Women's social worth is closely tied to their ability to bear children, rendering them more susceptible to scrutiny and blame for infertility,^6^^,^^13^^,^^43^^,^^48^ even when male factors are medically identified as the cause.^49^ Based on quantitative evidence, the level of stigma experienced varied among individuals based on their social class, and was particularly pronounced in those with a low level of education and occupational category.^15^ Moreover, experience of infertility stigma increased with individual's age and duration of infertility.^21^ This reflected in some narratives where individuals described mounting societal pressure as they aged.^5^^,^^22^^,^^48^

Marital status also plays a critical role in shaping experiences of stigma. Married couples, in particular, face significant pressure from family members and society at large.^8^^,^^9^^,^^20^^,^^28^^,^^34^^,^^45^^,^^46^ The perceived success of a marriage is often tied to the couple's ability to produce offspring. This societal metric of marital success places immense expectations on couples, especially women, to fulfil the role of procreation.^7^^,^^9^^,^^12^^,^^22^^,^^26^^,^^39^ As infertility persists over time, societal judgment intensifies, further marginalizing affected couples.^21^ In some cases, families intervened by proposing solutions that aligned with cultural norms but often undermined the autonomy of the individuals involved. Such interventions included remarriage, polygamy or even the suggestion of secretive extramarital procreation as a means to resolve the issue of childlessness.^8–10^^,^^29^^,^^39^^,^^43^^,^^47^^,^^48^^,^^50^

Intersecting stigma, where individuals are subjected to multiple layers of stigma simultaneously, was evident across many communities. Women with infertility were not only stigmatized for their inability to conceive, but also for their perceived association with contraceptive use and abortion.^6–9^^,^^20^^,^^23^^,^^31^^,^^35^^,^^36^^,^^40^^,^^46^ These additional stigmas were often based on unfounded suspicions, rumours and cultural assumptions rather than verifiable evidence. The societal stigma surrounding contraceptive use and abortion is deeply rooted in cultural and religious ideologies that frame reproductive autonomy as contradictory to divine will and traditional gender roles.^9^^,^^35^^,^^48^ In some communities, women suspected of having undergone abortions are harshly judged, with their infertility perceived as a punitive consequence of their prior reproductive choices.^9^^,^^48^ This narrative creates a perilous intersection of stigma, where infertility, already regarded as a personal failing, is exacerbated by moral condemnation linked to abortion.

### Manifestations of infertility stigma

Infertility stigma is manifested in various forms, encompassing several key domains and stigmatizing practices. These domains—experienced, perceived, internalized, anticipated, structural and associative stigma—although distinct, are interconnected and inherent in the ways stigma is practiced.

### Experienced stigma

Experienced stigma, the direct and lived reality of being treated unjustly or differently due to infertility, emerged as the most immediate and tangible form of stigma. Individuals facing infertility were routinely subjected to harsh accusations and pervasive stereotypes, which framed their condition as the result of personal failings or misconduct. These attributions included allegations of abortion,^9^^,^^20^^,^^31^^,^^35^^,^^36^^,^^40^ contraceptive use,^6–8^^,^^23^^,^^46^ sexual promiscuity,^10^^,^^31^ premarital sex^41^ and even involvement in occult practices, such as money rituals.^5^^,^^43^ For couples who had already borne children, especially women, the stigma could intensify, with accusations suggesting the child was conceived through infidelity.^48^

Stereotypes portraying infertility as a ‘curse’^10^^,^^12^^,^^31^ or a state of ‘incompleteness’^35^^,^^39^ deepens the marginalization of affected individuals. They are often labelled as ‘failure’,^37^^,^^40^^,^^49^ ‘useless’,^6^^,^^31^^,^^34^^,^^39^^,^^50^^,^^57^ ‘weak’^39^^,^^41^ or even linked to ‘witchcraft’,^10^^,^^22^^,^^36^^,^^43^^,^^44^^,^^47^^,^^51^ reinforcing negative perceptions. Childless women are particularly stigmatized, with their inability to conceive often framed as an emotional deficiency.^44^ They are often accused of harbouring jealousy toward mothers, being harmful to children and, as a result, are restricted from interacting with them.^13^^,^^22^ These women are also blamed for illness, misfortune or even death within their communities,^6^^,^^7^^,^^10^^,^^22^^,^^36^^,^^39^^,^^44^ and their infertility is a frequent subject of gossip,^6^^,^^12^^,^^22^^,^^23^^,^^28^^,^^33^^,^^39^^,^^41^^,^^46–48^^,^^51^ mockery^8^^,^^11^^,^^20^^,^^33^^,^^39^^,^^40^^,^^43^^,^^49^ and public scorn.^6^^,^^7^^,^^28^^,^^29^^,^^35^^,^^42^ In addition to verbal abuse, individuals with infertility are often dehumanized through derogatory terms, many of which are rooted in local dialects. For instance, terms like *mzidzi* (‘a pig who cannot reproduce’),^48^  *chizizi* (‘an egg that has failed to hatch during an incubation period of a chicken’)^48^ and *incabi* (‘a castrated cow’)^41^ are used to belittle and humiliate those struggling with infertility. These names not only insult the individual’s reproductive capacity, but also deny them their dignity, further entrenching their social marginalization.^48^

Discrimination also permeated the professional and social spheres. Individuals face challenges in securing employment, as they are perceived as unsuitable due to their inability to fulfil societal expectations tied to parenthood.^48^ Those already employed are subjected to unfair treatment, including being burdened with excessive overtime,^45^ based on the assumption that their lack of childcaring duties affords them more time and flexibility.^12^ In community settings, men with infertility face exclusion from inheritance rights,^10^ while women are denied basic necessities, such as food and money by their partners.^46^ Social exclusion is widespread, with a prevalence of 56% reported among couples in Ghana.^32^ Individuals with infertility are often ostracized by friends, family and even partners.^5^^,^^7–10^^,^^28^^,^^39^^,^^51^^,^^53^ They are barred from attending social events meant for ‘parents’,^7^^,^^8^^,^^20^^,^^46^ or if allowed to participate, their contributions are dismissed or ignored.^11^^,^^41^ Women, in particular, are excluded from childcare-related activities, reflecting deep-seated assumptions about their competence.^7^^,^^9^^,^^48^

In the context of marriage, the absence of children is often construed as a sign of divine disfavour, casting a shadow over the union.^22^ This cultural perception places intense pressure on couples, with women shouldering a disproportionate amount of blame for infertility. As a result, they frequently become the target of verbal abuse, physical violence and even threats of divorce. Stories of women being ‘beaten’ or ‘slapped’ by their husbands underscores the harsh physical repercussions of such blame.^7^^,^^40^^,^^46^ Beyond spousal tensions, couples are also subjected to immense pressure from their families, who often issue ultimatums demanding childbirth within specific time frames.^29^ Women who fail to fulfil these expectations face the looming threat of divorce,^8–10^^,^^29^^,^^39^^,^^47^^,^^48^^,^^50^ while men, too, are not immune from scrutiny. Their marital decisions, particularly their wives’ infertility, are questioned, with harsh judgments from both sides of the family.^36^^,^^39^^,^^40^^,^^51^

### Internalized stigma

Internalized stigma—where individuals absorb and accept negative stereotypes and societal beliefs about their condition—was widespread among persons with infertility in Africa. Many individuals internalized the belief that their infertility rendered them incomplete,^35^^,^^39^^,^^53^ leading to profound self-doubt about their femininity or masculinity.^10^^,^^39^^,^^50^ This self-perception often translated into feelings of worthlessness.^13^^,^^28^^,^^35^^,^^39^^,^^53^ For example, in the study by Annan-Frey et al.,^28^ one woman shared, ‘I sometimes feel God shouldn't have created me,’ reflecting a deep sense of despair. Feelings of inferiority are prominent among individuals with infertility. Many women experience a profound sense of personal failure, particularly those who feel they have not fulfilled their societal roles as wives and mothers.^39^^,^^50^^,^^51^

### Perceived and anticipated stigma

The prevalence of perceived stigma—an acute awareness of others’ negative attitudes towards one's condition—was reported in two studies, ranging from 51.3%^21^ to 64%.^15^ Individuals with infertility often exhibit a heightened sensitivity to social cues, interpreting actions or situations as reflective of societal disapproval. For instance, many women believed their infertility was a subject of gossip within their communities, even when they had not directly overheard any derogatory remarks.^7^ Social behaviours, such as avoidance by friends and family members, were also frequently perceived as signs of disdain or rejection.^9^^,^^35^^,^^39^

Anticipated stigma, where individuals expect negative treatment in the future due to their infertility, frequently coexists with perceived stigma. Many individuals, especially women, anticipate adverse reactions from others and consequently alter their behaviours to minimize exposure to potential judgment or discrimination.^7^^,^^10^^,^^12^^,^^22^^,^^33^^,^^35^^,^^39^^,^^43^^,^^45^ For instance, some avoid attending social gatherings where topics of childbearing might arise, fearing that their infertility will be scrutinized or questioned.^7^^,^^10^^,^^12^^,^^22^^,^^33^^,^^35^^,^^39^^,^^43^^,^^45^ Even when attending such events, some refrain from contributing to discussions due to fear of being insulted.^8^^,^^22^ Individuals hesitate to share their condition with friends or community members, fearing gossip, rejection or exclusion.^6^^,^^51^ This vigilance and self-protection often results in social withdrawal, reinforcing isolation and exacerbating the emotional toll of infertility.

### Structural stigma

Structural stigma is deeply rooted in religious, sociocultural norms and institutional practices, perpetuating the marginalization of individuals with infertility. Through a religious lens, infertility is often interpreted as divine punishment for moral failings or transgressions, fostering an environment of blame where individuals are held accountable for their condition.^10^^,^^31^^,^^44^ Moreover, cultural myths about infertility, such as the notion that it results from curses, reinforce fear and discrimination within communities, creating a hostile social atmosphere for individuals with infertility.^7^^,^^10^ Infertility is also perceived as a violation of traditional gender roles, with women disproportionately bearing the blame for childlessness.^42^^,^^46^ This normative expectation places immense social pressure on women, often subjecting them to emotional distress and public scrutiny.

Family dynamics also play a significant role in entrenching stigma.^42^ Family members often endorse discriminatory practices, such as encouraging men to seek new partners to resolve infertility.^8–10^^,^^29^^,^^47^ These actions not only reinforce gender-based blame but also exacerbate the isolation of women, eroding their social support networks and deepening their vulnerability. In healthcare settings, certain institutional practices, such as publicly announcing a patient’s condition, inadvertently intensifies infertility stigma.^23^ For instance, one participant recounted, ‘you go to the hospital, and they will announce that those with infertility should go to this consulting room, telling everybody of your problem. The next thing you see is that people are pointing fingers at you in town’.^23^ Such breaches of privacy not only expose individuals to community-wide scrutiny, but also deepen the layers of stigma they face, both within medical institutions and in their broader social environments.

### Associative stigma

The stigma of infertility extends beyond individuals to their close associates, particularly family members.^31^^,^^39^ In many communities, infertility is perceived not solely as an individual issue, but as a reflection of broader familial failings.^31^ For example, infertility is sometimes attributed to ancestral sins, creating a collective burden of shame for the families of affected individuals.^31^ In familial disputes, stigma is weaponized, with infertility framed as divine retribution affecting both the individual and their relatives. In one study, a participant described her mother's experience of stigma, recounting that ‘whenever disagreements arose between my mother and her relatives, they insulted her, saying that she had received her punishment through her daughter and labelled her as “cursed”’.^39^ This intersection of familial relationships and stigma underscores the far-reaching impact of infertility on both individuals and their social networks.

### Consequences of stigma

The stigma associated with infertility had far-reaching consequences on affected individuals. Psychological and emotional distress are among the most reported outcomes of infertility stigma. Individuals disclosed experiencing suicidal thoughts.^20^ Emotional distress and depression is pervasive, characterized by feelings of sadness and hopelessness.^11^^,^^12^^,^^43^ The societal devaluation of infertility leads individuals to internalize these negative perceptions, further eroding their self-worth.^28^^,^^35^^,^^39^^,^^53^ Many also experience an identity crisis, struggling to reconcile their self-image with societal expectations of parenthood.^39^^,^^50^ The emotional toll also manifests in physical symptoms, with individuals reporting difficulty sleeping and a loss of appetite due to stigma.^6^^,^^39^

Behavioural changes are evident as individuals seek to navigate the societal judgment tied to infertility. Many participants avoid seeking healthcare services, fearing stigmatization within clinic settings.^30^ To escape judgment, individuals often choose to conceal their infertility from family and friends, a decision that heightens their sense of isolation and disrupts their normal routines.^6^^,^^51^ Fear of societal judgment and internalized shame further drive some participants to withdraw from social interactions, severing connections with their broader social networks.^7^^,^^10^^,^^22^^,^^35^^,^^43^^,^^45^ This withdrawal not only limits their emotional support, but also exacerbates their sense of loneliness and exclusion.^9^^,^^35^

Infertility stigma places immense strain on personal relationships, particularly with spouses and extended family members. Divorce or separation is a common outcome, with stigma often cited as a key factor contributing to the dissolution of marriages.^8^^,^^13^^,^^22^^,^^46^ For couples who remain together, infertility-related stigma frequently causes tension and conflict, weakening the marital bond and eroding trust.^29^^,^^38^ Mistrust also extends beyond familial relationships to healthcare providers, as individuals fear breaches of confidentiality regarding their infertility status.^12^ This mistrust reinforces a reluctance to seek medical care and deepens their emotional isolation.^30^

## Discussion

### Summary of evidence

This systematic review and meta-synthesis identified four overarching themes characterizing infertility stigma in Africa: mechanisms driving or facilitating infertility stigma, stigma marking, manifestations and consequences. Infertility stigma is deeply rooted in misconceptions, sociocultural norms, religious beliefs and institutional practices, where childlessness is viewed as a personal failure and a violation of traditional gender roles, especially for women. Stigma marking was evident, with women bearing the brunt of societal blame, regardless of the cause of infertility, and those from lower socio-economic backgrounds and with extended periods of childlessness facing intensified stigma. Infertility stigma manifested across several domains, including experienced, perceived, internalized, anticipated, structural and associative stigma. Individuals encountering these stigmas endured ridicule, discrimination and social exclusion, with some even subjected to physical violence or complete isolation. These encounters had far-reaching consequences, including psychological distress, behavioural changes, relational strain and diminished quality of life.

### Uniquely African dimensions of infertility stigma

While infertility stigma shares certain universal traits, the African context reveals distinct cultural and social dimensions that amplify its impact. In diverse settings, infertility stigma is often rooted in gendered expectations surrounding procreation. For example, in Utah (USA), infertility in women was linked to unmet societal and familial roles, leading to feelings of inadequacy.^58^ Similarly, in Asia, women with infertility face ostracization and verbal or physical abuse,^59^^,^^60^ paralleling some findings from African settings.

In Africa, however, infertility stigma is mostly intertwined with collective identities and cultural rituals, heightening its visibility and societal impact. Language plays a significant role in perpetuating this stigma. Many African dialects include derogatory terms for childlessness, serving as potent labels that reinforce stigmatization in daily interactions.^41^^,^^42^^,^^48^ This linguistic specificity contrasts with the more subtle or generalized expressions of infertility stigma observed in other regions. Furthermore, infertility in African contexts is often interpreted through spiritual frameworks, with beliefs attributing infertility to curses, witchcraft or divine punishment. These beliefs not only ostracize affected individuals, but also pave the way for exploitative practices such as expensive and ineffective rituals. While spiritual interpretations of infertility exist globally, their pervasive influence on societal attitudes and behaviours is especially prominent in Africa.^61^ The communal nature of African societies also differentiates the experience of infertility stigma. Here, infertility is frequently viewed as a failure of the family or lineage, not just the individual.^31^^,^^39^ This perspective creates unique pressures, such as familial arrangements for a man to take a second wife.^39^^,^^47^ Distinct cultural symbols further embed infertility stigma within African societies. Examples include the symbolic use of the doek worn by childless women^40^ or the practice of dressing deceased infertile men in women's clothing during funerals.^10^ These culturally specific markers intensify the stigma, making it uniquely pervasive in African contexts.

### Evidence gaps and opportunities for future research

Among the 48 identified studies, only a small fraction (5 studies) employed quantitative methodologies, with just 1 specifically utilizing a scale centred on infertility stigma. This scarcity points to a critical need for standardized and validated tools capable of capturing the complex dynamics of infertility stigma, especially within the African context, where cultural and social factors uniquely shape stigma experiences.

Several gaps emerged from the evidence synthesis. First, there is limited exploration of healthcare providers’ roles, attitudes and practices that may exacerbate stigma. This gap is largely tied to the broader issue of Africa's shortage of infertility services.^62^ This underscores the importance of investigating barriers to expanding infertility care and exploring strategies for integrating infertility services into existing healthcare systems. In the interim, it is crucial to examine how healthcare providers’ attitudes and practices impact patient experiences in areas where infertility services are available and whether targeted training interventions can help reduce stigma and improve care delivery. Second, gender-specific insights are also lacking, with minimal focus on men's experiences and how societal expectations around masculinity shape infertility stigma. Third, there is a lack of information on effective coping mechanisms or interventions to address infertility stigma and mitigate its impact on individuals. Fourth, longitudinal studies examining the long-term impact and social trajectories of individuals facing infertility stigma are lacking. Addressing these gaps would provide a stronger foundation for developing targeted, culturally responsive interventions to support those affected by infertility stigma in Africa.

### Strengths and limitations

To the best of our knowledge, this review is the first of its kind to provide an in-depth exploration of infertility stigma within African settings, contributing valuable insights to the global reproductive health discourse. However, it is not without its limitations. First, it focused on published peer-reviewed literature, which may exclude valuable perspectives from unpublished sources. Additionally, the inclusion criterion was restricted to studies published in English, potentially omitting important research from regions where other languages predominate. Furthermore, although the study captures a wide array of experiences related to infertility stigma across diverse African communities, it does not account for the potential regional variations within the continent. Given Africa's vast cultural and ethnic diversity, beliefs and practices surrounding infertility may differ significantly across regions. Future research should focus on comparing these regional differences to offer a more nuanced and comprehensive understanding of infertility stigma in Africa.

## Conclusions

This systematic review and meta-synthesis present a framework that consolidates evidence of infertility stigma in Africa. The framework's contribution lies in its ability to bridge the gap between empirical findings and actionable insights. For instance, it identifies critical gaps in the literature, such as the lack of standardized tools to measure infertility stigma, limited focus on men's experiences and the absence of interventions tailored to African contexts. These gaps present opportunities for future research to develop culturally sensitive scales, explore gender-specific experiences and design studies to assess the long-term impacts of stigma. From a policy and programmatic perspective, the framework calls for multifaceted interventions that address the structural and cultural drivers of stigma. This includes community-based education campaigns to dispel myths about infertility, training programs for healthcare providers to reduce stigmatizing practices and the integration of infertility services into existing reproductive health systems. The framework also emphasizes the need for policies that protect the rights of individuals with infertility, such as safeguarding against workplace discrimination.

## Supplementary Material

ihaf060_Supplemental_File

## Data Availability

The data that support the findings of this study are available in the article and its supplemental files.
