# Supplementary material for: Experience of infertility-related stigma in Africa: a systematic review and mixed methods meta-synthesis
Source: Int Health. 2025 May 27;17(6):903–13. doi: 10.1093/inthealth/ihaf060 (PMC12585573; doi:10.1093/inthealth/ihaf060)
Supplement: ihaf060_Supplemental_File [file ihaf060_supplemental_file.docx]

**SUPPLEMENTARY FILE**

Items

Appendix A: Database search strategy

Appendix B: Study quality appraisal

**Appendix A**

PubMed search strategy

| Concept | Search string |
| --- | --- |
| #1 Infertility | "Infertility"[Mesh] OR Infertility OR infertile OR unfertile OR subfertility OR sub-fertility OR sterile OR sterility OR "reproductive sterility" OR infecund OR infecundity OR barren OR barrenness OR unproductive OR unfruitful OR childless OR childlessness OR impoten* |
| #2 Stigma | "Social Stigma"[Mesh] OR "Social Discrimination"[Mesh] OR stigma OR "social stigma" OR self-stigma OR "perceived stigma" OR "anticipated stigma" OR stereotyp* OR discriminat* OR "social discrimination" OR prejudice OR sham* OR blam* OR label* OR misjudge* OR "negative self-Image" OR devalue* OR marginaliz* OR "social exclusion" |
| #3 Africa | "Africa"[Mesh] OR Africa OR Algeria OR Angola OR Benin OR Botswana OR "Burkina Faso" OR Burundi OR "Cabo Verde" OR Cameroon OR "Central African Republic" OR Chad OR Comoros OR Congo OR "Democratic Republic of Congo" OR "Cote d'Ivoire" OR Djibouti OR Egypt OR "Equatorial Guinea" OR Eritrea OR Eswatini OR Ethiopia OR Gabon OR Gambia OR Ghana OR Guinea OR Guinea-Bissau OR Kenya OR Lesotho OR Liberia OR Libya OR Madagascar OR Malawi OR Mali OR Mauritania OR Mauritius OR Morocco OR Mozambique OR Namibia OR Niger OR Nigeria OR Rwanda OR "Sao Tome and Principe" OR Senegal OR Seychelles OR "Sierra Leone" OR Somalia OR "South Africa" OR "South Sudan" OR Sudan OR Tanzania OR Togo OR Tunisia OR Uganda OR Zambia OR Zimbabwe |
| #4 | #1 AND #2 AND #3 |

Medline search strategy

| Concept | Search string |
| --- | --- |
| #1 Infertility | exp Infertility/ OR (Infertility OR infertile OR unfertile OR subfertility OR sub-fertility OR sterile OR sterility OR "reproductive sterility" OR infecund OR infecundity OR barren OR barrenness OR unproductive OR unfruitful OR childless OR childlessness OR impoten*).mp. |
| #2 Stigma | exp Social Stigma/ OR exp Stereotyping/ OR exp Prejudice/ OR exp Social Discrimination/ OR (stigma OR "social stigma" OR self-stigma OR "perceived stigma" OR "anticipated stigma" OR stereotyp* OR discriminat* OR "social discrimination" OR prejudice OR sham* OR blam* OR label* OR misjudge* OR "negative self-Image" OR devalue* OR marginaliz* OR "social exclusion").mp. |
| #3 Africa | exp Africa/ OR (Africa OR Algeria OR Angola OR Benin OR Botswana OR "Burkina Faso" OR Burundi OR "Cabo Verde" OR Cameroon OR "Central African Republic" OR Chad OR Comoros OR Congo OR "Democratic Republic of Congo" OR "Cote d'Ivoire" OR Djibouti OR Egypt OR "Equatorial Guinea" OR Eritrea OR Eswatini OR Ethiopia OR Gabon OR Gambia OR Ghana OR Guinea OR Guinea-Bissau OR Kenya OR Lesotho OR Liberia OR Libya OR Madagascar OR Malawi OR Mali OR Mauritania OR Mauritius OR Morocco OR Mozambique OR Namibia OR Niger OR Nigeria OR Rwanda OR "Sao Tome and Principe" OR Senegal OR Seychelles OR "Sierra Leone" OR Somalia OR "South Africa" OR "South Sudan" OR Sudan OR Tanzania OR Togo OR Tunisia OR Uganda OR Zambia OR Zimbabwe).mp. |
| #4 | #1 AND #2 AND #3 |

CINAHL search strategy

| Concept | Search string |
| --- | --- |
| #1 Infertility | (MH "Infertility+") OR Infertility OR infertile OR unfertile OR subfertility OR sub-fertility OR sterile OR sterility OR "reproductive sterility" OR infecund OR infecundity OR barren OR barrenness OR unproductive OR unfruitful OR childless OR childlessness OR impoten* |
| #2 Stigma | (MH "Stigma") OR (MH "Discrimination+") OR stigma OR "social stigma" OR self-stigma OR "perceived stigma" OR "anticipated stigma" OR stereotyp* OR discriminat* OR "social discrimination" OR prejudice OR sham* OR blam* OR label* OR misjudge* OR "negative self-Image" OR devalue* OR marginaliz* OR "social exclusion" |
| #3 Africa | (MH "Africa+") OR Africa OR Algeria OR Angola OR Benin OR Botswana OR "Burkina Faso" OR Burundi OR "Cabo Verde" OR Cameroon OR "Central African Republic" OR Chad OR Comoros OR Congo OR "Democratic Republic of Congo" OR "Cote d'Ivoire" OR Djibouti OR Egypt OR "Equatorial Guinea" OR Eritrea OR Eswatini OR Ethiopia OR Gabon OR Gambia OR Ghana OR Guinea OR Guinea-Bissau OR Kenya OR Lesotho OR Liberia OR Libya OR Madagascar OR Malawi OR Mali OR Mauritania OR Mauritius OR Morocco OR Mozambique OR Namibia OR Niger OR Nigeria OR Rwanda OR "Sao Tome and Principe" OR Senegal OR Seychelles OR "Sierra Leone" OR Somalia OR "South Africa" OR "South Sudan" OR Sudan OR Tanzania OR Togo OR Tunisia OR Uganda OR Zambia OR Zimbabwe |
| #4 | #1 AND #2 AND #3 |

PsycINFO search strategy

| Concept | Search string |
| --- | --- |
| #1 Infertility | exp Infertility/ OR exp Sterility/ OR (Infertility OR infertile OR unfertile OR subfertility OR sub-fertility OR sterile OR sterility OR "reproductive sterility" OR infecund OR infecundity OR barren OR barrenness OR unproductive OR unfruitful OR childless OR childlessness OR impoten*).mp. |
| #2 Stigma | exp Stigma/ OR exp Self-Stigma/ OR exp Stereotyped Attitudes/ OR exp Prejudice/ OR exp Discrimination/ OR (stigma OR "social stigma" OR self-stigma OR "perceived stigma" OR "anticipated stigma" OR stereotyp* OR discriminat* OR "social discrimination" OR prejudice OR sham* OR blam* OR label* OR misjudge* OR "negative self-Image" OR devalue* OR marginaliz* OR "social exclusion").mp. |
| #3 Africa | (Africa OR Algeria OR Angola OR Benin OR Botswana OR "Burkina Faso" OR Burundi OR "Cabo Verde" OR Cameroon OR "Central African Republic" OR Chad OR Comoros OR Congo OR "Democratic Republic of Congo" OR "Cote d'Ivoire" OR Djibouti OR Egypt OR "Equatorial Guinea" OR Eritrea OR Eswatini OR Ethiopia OR Gabon OR Gambia OR Ghana OR Guinea OR Guinea-Bissau OR Kenya OR Lesotho OR Liberia OR Libya OR Madagascar OR Malawi OR Mali OR Mauritania OR Mauritius OR Morocco OR Mozambique OR Namibia OR Niger OR Nigeria OR Rwanda OR "Sao Tome and Principe" OR Senegal OR Seychelles OR "Sierra Leone" OR Somalia OR "South Africa" OR "South Sudan" OR Sudan OR Tanzania OR Togo OR Tunisia OR Uganda OR Zambia OR Zimbabwe).mp. |
| #4 | #1 AND #2 AND #3 |

Global Health search strategy

| Concept | Search string |
| --- | --- |
| #1 Infertility | exp Infertility/ OR exp Sterility/ OR (Infertility OR infertile OR unfertile OR subfertility OR sub-fertility OR sterile OR sterility OR "reproductive sterility" OR infecund OR infecundity OR barren OR barrenness OR unproductive OR unfruitful OR childless OR childlessness OR impoten*).mp. |
| #2 Stigma | exp Stigma/ OR exp social stigma/ OR exp discrimination/ OR (stigma OR "social stigma" OR self-stigma OR "perceived stigma" OR "anticipated stigma" OR stereotyp* OR discriminat* OR "social discrimination" OR prejudice OR sham* OR blam* OR label* OR misjudge* OR "negative self-Image" OR devalue* OR marginaliz* OR "social exclusion").mp. |
| #3 Africa | exp Africa/ OR (Africa OR Algeria OR Angola OR Benin OR Botswana OR "Burkina Faso" OR Burundi OR "Cabo Verde" OR Cameroon OR "Central African Republic" OR Chad OR Comoros OR Congo OR "Democratic Republic of Congo" OR "Cote d'Ivoire" OR Djibouti OR Egypt OR "Equatorial Guinea" OR Eritrea OR Eswatini OR Ethiopia OR Gabon OR Gambia OR Ghana OR Guinea OR Guinea-Bissau OR Kenya OR Lesotho OR Liberia OR Libya OR Madagascar OR Malawi OR Mali OR Mauritania OR Mauritius OR Morocco OR Mozambique OR Namibia OR Niger OR Nigeria OR Rwanda OR "Sao Tome and Principe" OR Senegal OR Seychelles OR "Sierra Leone" OR Somalia OR "South Africa" OR "South Sudan" OR Sudan OR Tanzania OR Togo OR Tunisia OR Uganda OR Zambia OR Zimbabwe).mp. |
| #4 | #1 AND #2 AND #3 |

Scopus search strategy

| Concept | Search string |
| --- | --- |
| #1 Infertility | TITLE-ABS-KEY (Infertility OR infertile OR unfertile OR subfertility OR sub-fertility OR sterile OR sterility OR "reproductive sterility" OR infecund OR infecundity OR barren OR barrenness OR unproductive OR unfruitful OR childless OR childlessness OR impoten*) |
| #2 Stigma | TITLE-ABS-KEY (stigma OR "social stigma" OR self-stigma OR "perceived stigma" OR "anticipated stigma" OR stereotyp* OR discriminat* OR "social discrimination" OR prejudice OR sham* OR blam* OR label* OR misjudge* OR "negative self-Image" OR devalue* OR marginaliz* OR "social exclusion") |
| #3 Africa | TITLE-ABS-KEY (Africa OR Algeria OR Angola OR Benin OR Botswana OR "Burkina Faso" OR Burundi OR "Cabo Verde" OR Cameroon OR "Central African Republic" OR Chad OR Comoros OR Congo OR "Democratic Republic of Congo" OR "Cote d'Ivoire" OR Djibouti OR Egypt OR "Equatorial Guinea" OR Eritrea OR Eswatini OR Ethiopia OR Gabon OR Gambia OR Ghana OR Guinea OR Guinea-Bissau OR Kenya OR Lesotho OR Liberia OR Libya OR Madagascar OR Malawi OR Mali OR Mauritania OR Mauritius OR Morocco OR Mozambique OR Namibia OR Niger OR Nigeria OR Rwanda OR "Sao Tome and Principe" OR Senegal OR Seychelles OR "Sierra Leone" OR Somalia OR "South Africa" OR "South Sudan" OR Sudan OR Tanzania OR Togo OR Tunisia OR Uganda OR Zambia OR Zimbabwe) |
| #4 | #1 AND #2 AND #3 |

Web of Science

| Concept | Search string |
| --- | --- |
| #1 Infertility | Topic (Infertility OR infertile OR unfertile OR subfertility OR sub-fertility OR sterile OR sterility OR "reproductive sterility" OR infecund OR infecundity OR barren OR barrenness OR unproductive OR unfruitful OR childless OR childlessness OR impoten*) |
| #2 Stigma | Topic (stigma OR "social stigma" OR self-stigma OR "perceived stigma" OR "anticipated stigma" OR stereotyp* OR discriminat* OR "social discrimination" OR prejudice OR sham* OR blam* OR label* OR misjudge* OR "negative self-Image" OR devalue* OR marginaliz* OR "social exclusion") |
| #3 Africa | Topic (Africa OR Algeria OR Angola OR Benin OR Botswana OR "Burkina Faso" OR Burundi OR "Cabo Verde" OR Cameroon OR "Central African Republic" OR Chad OR Comoros OR Congo OR "Democratic Republic of Congo" OR "Cote d'Ivoire" OR Djibouti OR Egypt OR "Equatorial Guinea" OR Eritrea OR Eswatini OR Ethiopia OR Gabon OR Gambia OR Ghana OR Guinea OR Guinea-Bissau OR Kenya OR Lesotho OR Liberia OR Libya OR Madagascar OR Malawi OR Mali OR Mauritania OR Mauritius OR Morocco OR Mozambique OR Namibia OR Niger OR Nigeria OR Rwanda OR "Sao Tome and Principe" OR Senegal OR Seychelles OR "Sierra Leone" OR Somalia OR "South Africa" OR "South Sudan" OR Sudan OR Tanzania OR Togo OR Tunisia OR Uganda OR Zambia OR Zimbabwe) |
| #4 | #1 AND #2 AND #3 |

**Appendix B**

Result of the quality assessment of studies using the Mixed Methods Appraisal Tool (MMAT)

Quantitative studies

| Author (year) | Are there clear research questions? | Do the collected data allow to address the research questions? | Is the sampling strategy relevant to address the research question? | Is the sample representative of the target population? | Are the measurements appropriate? | Is the risk of nonresponse bias low? | Is the statistical analysis appropriate to answer the research question? |
| --- | --- | --- | --- | --- | --- | --- | --- |
| Kyei (2024) | Yes | Yes | No | Yes | Yes | Can’t tell | Yes |
| Naab (2013) | Yes | Yes | Can’t tell | Can’t tell | Yes | Can’t tell | Yes |
| Donkor (2007) | Yes | Yes | Can’t tell | Yes | Yes | Yes | Yes |
| Van Rooij (2021) | Yes | Yes | No | Can’t tell | Yes | Can’t tell | Yes |
| Anokye (2017) | Yes | Yes | Yes | Can’t tell | Can’t tell | Yes | Can’t tell |

Qualitative studies

| Author (year) | Are there clear research questions? | Do the collected data allow to address the research questions? | Is the qualitative approach appropriate to answer the research question? | Are the qualitative data collection methods adequate to address the research question? | Are the findings adequately derived from the data? | Is the interpretation of results sufficiently substantiated by data? | Is there coherence between qualitative data sources, collection, analysis and interpretation? |
| --- | --- | --- | --- | --- | --- | --- | --- |
| Kyei (2021) | Yes | Yes | Yes | Yes | Yes | Yes | Yes |
| Tabong (2013) a | Yes | Yes | Yes | Yes | Yes | Yes | Yes |
| Tabong (2013) b | Yes | Yes | Yes | Yes | Yes | Yes | Yes |
| Naab (2018) | Yes | Yes | Yes | Yes | Yes | Yes | Yes |
| Naab (2019) | Yes | Yes | Yes | Yes | Yes | Yes | Yes |
| Donkor  (2017) | Yes | Yes | Yes | Yes | Yes | Yes | Yes |
| Kuug (2023) | Yes | Yes | Yes | Yes | Yes | Yes | Yes |
| Diallo (2024) | Yes | Yes | Yes | Yes | Yes | Yes | Yes |
| Fledderjohann  (2012) | Yes | Yes | Yes | Yes | Yes | Yes | Yes |
| Annan-Frey (2023) | Yes | Yes | Yes | Yes | Yes | Yes | Yes |
| Ofosu-Budu (2020) | Yes | Yes | Yes | Yes | Yes | Yes | Yes |
| Adane (2024) a | Yes | Yes | Yes | Yes | Yes | Yes | Yes |
| Adane (2024) b | Yes | Yes | Yes | Yes | Yes | Yes | Yes |
| Dierickx  (2018) | Yes | Yes | Yes | Yes | Yes | Yes | Yes |
| Dierickx  (2021) | Yes | Yes | Yes | Yes | Yes | Yes | Yes |
| Dierickx  (2022) | Yes | Yes | Yes | Yes | Yes | Yes | Yes |
| Mussie  (2017) | Yes | Yes | Yes | Yes | Can’t tell | Yes | Yes |
| Moyo (2013) | Yes | Yes | Yes | Yes | Can’t tell | Yes | Yes |
| Bornstein (2020) | Yes | Yes | Yes | Yes | Yes | Yes | Yes |
| Weinger (2009) | No | Can’t tell | Can’t tell | Yes | Yes | Yes | Yes |
| Esan (2022) | Yes | Yes | Yes | Yes | Yes | Yes | Yes |
| Hiadzi (2022) | Yes | Yes | Yes | Yes | Yes | Yes | Yes |
| Dyer (2002) | Yes | Yes | Yes | Yes | Yes | Yes | Yes |
| Dyer (2004) | Yes | Yes | Yes | Yes | Yes | Yes | Yes |
| Hollos (2014) | Yes | Yes | Can’t tell | Yes | Can’t tell | Can’t tell | No |
| Hollos (2008) | Yes | Yes | Can’t tell | Yes | Can’t tell | Can’t tell | No |
| Arhin (2022) | Yes | Yes | Yes | Yes | Yes | Yes | Yes |
| Elwell  (2022) | Yes | Yes | Yes | Yes | Can’t tell | Yes | Yes |
| Mabasa (2002) | Yes | Yes | Yes | Yes | Yes | Yes | Yes |
| Okantey (2021) | Yes | Yes | Yes | Yes | Yes | Yes | Yes |
| Asiimwe  (2022) | Yes | Yes | Yes | Yes | Yes | Yes | Yes |
| Dimka (2013) | Yes | Yes | Yes | Yes | Yes | Yes | Yes |
| Nieuwenhuis  (2009) | Yes | Yes | Yes | Yes | Yes | Yes | Yes |
| Nguimfack  (2016) | Yes | Yes | Yes | Yes | Yes | Yes | Yes |
| Njogu (2022) | Yes | Yes | Yes | Yes | Yes | Yes | Yes |
| Mashaah  (2024) | Yes | Yes | Yes | Yes | Yes | Yes | Yes |
| Ingwani (2021) | Yes | Yes | Yes | Yes | Can’t tell | Yes | Yes |
| Ochieng' (2020) | Yes | Yes | Yes | Yes | Can’t tell | Yes | Yes |
| Pratt (2025) | Yes | Yes | Yes | Yes | Yes | Yes | Yes |
| Benbella (2025) | Yes | Yes | Yes | Yes | Yes | Yes | Yes |

Mixed method

| Author (year) | Are there clear research questions? | Do the collected data allow to address the research questions? | Is there an adequate rationale for using a mixed methods design to address the research question? | Are the different components of the study effectively integrated to answer the research question? | Are the outputs of the integration of qualitative and quantitative components adequately interpreted? | Are divergences and inconsistencies between quantitative and qualitative results adequately addressed? | Do the different components of the study adhere to the quality criteria of each tradition of the methods involved? |
| --- | --- | --- | --- | --- | --- | --- | --- |
| Hess (2018) | Yes | Yes | Yes | Yes | Yes | No | No |
| Olowokere (2022) | Yes | Yes | Yes | Yes | Yes | No | No |
| Dhont  (2011) | Yes | Yes | Yes | No | Can’t tell | No | No |
